# Role of Regulatory T Cells in Pathogenesis and Biological Therapy of Multiple Sclerosis

**DOI:** 10.1155/2013/963748

**Published:** 2013-05-12

**Authors:** Milan Buc

**Affiliations:** Department of Immunology, Comenius University School of Medicine, 813 72 Bratislava, Slovakia

## Abstract

Multiple sclerosis (MS) is an inflammatory disease in which the myelin sheaths around the axons of the brain and spinal cord are damaged, leading to demyelination and scarring as well as a broad spectrum of signs and symptoms. It is caused by an autoimmune response to self-antigens in a genetically susceptible individual induced by unknown environmental factors. Principal cells of the immune system that drive the immunopathological processes are T cells, especially of T_H_1 and T_H_17 subsets. However, in recent years, it was disclosed that regulatory T cells took part in, too. Subsequently, there was endeavour to develop ways how to re-establish their physiological functions. In this review, we describe known mechanisms of action, efficacy, and side-effects of contemporary and emerging MS immunotherapeutical agents on Treg cells and other cells of the immune system involved in the immunopathogenesis of the disease. Furthermore, we discuss how laboratory immunology can offer physicians its help in the diagnosis process and decisions what kind of biological therapy should be used.

## 1. Introduction

The physiological function of the immune system is defence against external and internal violators of integrity of the organism. External “enemies” are represented mainly by germs; those of internal origin belong especially to potentially malignant cells that appear in our organisms as a result of the breakdown of their replication mechanisms. Another important biological function of the immune system is the prevention of autoreactive T and B cells activation, respectively, which potentially represent a threat of autoimmune diseases induction. To avoid this possibility, mechanisms of recessive (central) and dominant (peripheral) tolerance were developed. Recessive tolerance is based on deletion of autoreactive T and B cells in the thymus or in the bone marrow, respectively, during the process of their maturation in these primary lymphoid organs [1, 2]. Like other biological systems, the mechanisms of the recessive tolerance are not 100% effective, and a part of autoreactive lymphocytes escape their demise and enter the periphery, the secondary lymphoid organs. Here, when they encounter autoantigens, cross-reactive antigens or when a dysregulation of the immune system develops, they can be activated and induce autoimmune processes. Mechanisms of dominant tolerance mediated mainly by regulatory T cells (Treg) prevent this eventuality. By contacting with autoreactive lymphocytes directly or indirectly, especially by synthesis of immunosuppressive cytokines, Treg cells prevent their activation or suppress their effector activity [1, 2].

## 2. Regulatory T and B Cells

Regulatory T cells are divided into two populations:natural and induced (adaptive). Natural Treg cells (nTreg) represent an independent population, such as B lymphocytes, NK, and NKT cells. On the other hand, induced regulatory T cells (iTreg) is a population that develops during the immune response only; they represent a subset of CD4^+^ T helper cells [3, 4].

Natural regulatory T cells differentiate in the thymus. To develop, their T cell receptor (TCR*αβ*) must recognise peptides originating from self-antigens presented by HLA molecules in membranes of dendritic cells (DC); the recognition is highly affinitive. Moreover, costimulatory interactions between CD28 (nTreg) and CD80/CD86 (DC) as well as signalling processes induced by IL-2 or IL-15 are indispensable for their differentiation [3, 5]. 

Signalling processes result in formation of various transcription factors such as BLIMP-1, IRF4, transcription factors of the FOXO family, STAT5, and especially FOXP3 (forkhead box P3). It binds DNA and acts as a transcriptional activator/repressor by recruiting deacetylases as well as histone acetyltransferases. FOXP3 is crucial for the function of the nTreg cells. Humans with mutations in the FOXP3 gene (Xp11.23-q13.3) suffer from a severe autoimmune disorder known as IPEX syndrome (immune dysregulation, polyendocrinopathy, enteropathy, and X-linked), which manifests in lymphoproliferation, multiorgan lymphocytic infiltration, and systemic autoimmune inflammation; the disease is incompatible with life [6, 7].

nTreg cells comprise 5–10% of all CD4^+^CD8^−^ thymocytes and in the periphery they represent approximately 10% out of the whole population of CD4^+^ T cells. nTreg cells are long-living cells and IL-2 is essential for their peripheral maintenance as proved by its neutralisation, which results in reduction of nTreg cells counts and sensitisation to autoimmunity [8–10]. They are characterised by several membrane molecules. Characteristically, they express CD4, CD25, CD5, CD49d, CD69, CD103, CD152 (CTLA-4), and CD357 (GITR). More typical signs are low expression of CD127 (alpha chain of IL-7 receptor) and high expression of neuropilin [3, 11].

nTreg cells are anergic, that is they do not respond to *in vitro *stimulation by anti-CD3 monoclonal antibodies, phytohaemagglutinin, or allogeneic cells and do not produce IL-2. However, they downregulate activities of T cells, B, NK, NKT, and dendritic cells. The mechanisms they use include the production of immunosuppressive cytokines (IL-10, TGF-*β*, and IL-35), induction of apoptotic processes and metabolic alterations in target cells, and interference with maturation of dendritic cells [12–14].

Induced regulatory T cells differentiate from naïve CD4^+^ T helper cells during the immune response. They develop especially in a mucosal environment; their differentiation is best elucidated in the intestine. iTreg cells appear after naïve CD4^+^ T-lymphocytes are exposed to antigens, TGF-*β*, IL-2, and retinoic acid produced by DCs from vitamin A [2, 4, 15]. iTreg cells differentiate under suboptimal TCR stimulation without the necessity of CD28 costimulation. Like the nTreg cells, they also need FOXP3 for their development; however, a different part of proximal conserved noncoding sequences in its locus (CNS1) and different transcription factors (esp. NFAT). No systemic disease similar to IPEX happens when there is a deficiency of iTreg cells (in experimental animals); however, inflammatory processes of mucosal surfaces develop (colitis, asthma bronchiale) [16–18]. Initially, iTreg and nTreg cells were difficult to distinguish from one another. The problem was resolved only recently when it was found that iTreg, unlike nTreg, expressed much lower quantities of neuropilin [19]. iTreg cells suppress activities of target cells in a similar way like nTreg cells [4, 15].

What is the basic difference between nTreg and iTreg cells? Both suppress activities of cells of the immune system. nTreg cells principally downregulate activities of autoreactive T cells, that is, those that escaped from their demise in the thymus, for example, those that recognise myelin basic protein (MBP). iTreg cells restrict activities of effector T cells induced during the immune response to antigens, for example, the ones that are present in our intestines as commensals. nTreg and iTreg cells are not interchangeable in their activities; they complement each other [16].

B cells, to our surprise, can act as immunosuppressive cells too. Their downregulatory activity is mediated either by a direct contact with targets cells or by TGF-*β*, and especially by IL-10, or they can induce apoptosis of activated T cells. Their characteristic membrane molecules are known in mice, not yet in humans. They are designated as Breg or B10 cells [20–22]. 

## 3. Immunopathogenesis of Multiple Sclerosis and Neuromyelitis Optica

Multiple sclerosis (MS) is a characteristic autoimmune disease. In short, MS is characterised by demyelination process in the brain and the spinal cord; the peripheral nervous system is rarely involved. Aetiology of the disease is still unknown, most likely MS occurs because of some combination of genetic, environmental, and infectious agents, among them EBV, CMV, HBV, HSV, human herpetic viruses 6, or 7, measles viruses, coronaviruses, and others. A relationship between viruses and MS is supported by observations that viral infections frequently precede bouts of the disease. It is possible that IFN-*γ*, which is produced during the infection, triggers immunopathological events resulting in demyelination [23]. The viruses may possess proteins that resemble the myelin antigens and by the mechanisms of molecular mimicry activate autoreactive T cells. Environmental factors like insufficient supply of vitamin D seem to support MS development [24, 25]. Furthermore, MS prevalence rates may be influenced by the socioeconomic changes in previous decades, which are related to industrialisation, urban living, pollution, occupational exposures to solvents, changes in diet, smoking habits, and so forth.

**Figure 1:**
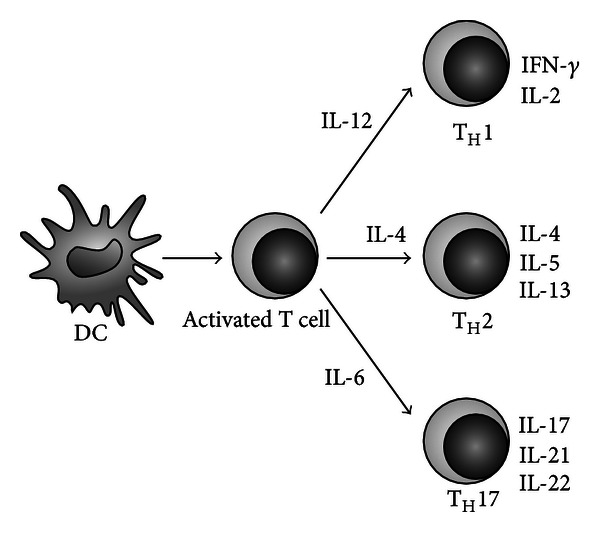
Differentiation of naïve T helper cells into particular subsets. T helper lymphocytes leaving the thymus (naïve or T_H_0) are not yet fully differentiated to perform their specific functions in peripheral lymphoid tissues. They are endowed of these properties in the process of their interactions with dendritic cells (DCs) that engulf, process, and present antigens to them. Moreover, DCs in dependence of the processed antigens produce different cytokines. If DCs produce IL-12, naïve T cells polarise into the T_H_1 subset, if IL-4 into the T_H_2 subset and eventually, if DCs synthesise IL-6, naïve T helper cells will become the T_H_17 cells.

Immunopathological processes start by activation of autoreactive T cells in the periphery; they belong to T_H_1, and to T_H_17 subsets (Figure 1) [23, 26]. Activated T cells subsequently upregulate the expression of adhesive molecules which enables them to adhere to their counterparts in membranes of endothelial cells of the central nervous system (CNS). LFA-1 and *α*4*β*1-integrins in T cells and ICAM-1 and VCAM-1 in endothelial cells mediate these interactions. VCAM-1 is constitutively expressed and its expression is substantially upregulated by stimulation of cells by cytokines. This mechanism of transmigration into the CNS parenchyma is used mainly by T_H_1 cells. T_H_17 cells prefer interaction between their chemokine receptors CCR6 and CCL20 ligands, which are also constitutively expressed in small quantities in membranes of endothelial cells [27, 28]. Likewise, B cells migration across the blood-brain barrier (BBB) is mediated by interaction between their CCR7 and CCL19 in the brain [28].

Upon entering the CNS, T cells are reactivated by local and infiltrating antigen presenting cells (i.e. dendritic cells and macrophages), which present peptides originated from myelin by their HLA class II molecules to T cells [29–31]. The activated T cells migrate into the parenchyma and produce proinflammatory cytokines (esp. IFN-*γ*, IL-17), which themselves may damage myelin [26]. However, more importantly, they activate microglial cells, which are thought to be the main cells responsible for lesional and perilesional axon killing. They, by the synthesis of cytokines (IL-12, IL-23, osteopontin), reactive oxygen, and nitrogen intermediate products, further contribute to the damage of myelin sheaths resulting in impaired nerve conduction [26, 30].

Studies on experimental autoimmune encephalomyelitis (EAE) induction suggested that only T_H_1 cells could access the CNS initially and this facilitated subsequent recruitment of T_H_17 cells [32]. They produced various cytokines, especially IL-17 and they themselves killed human neurons and promoted central nervous system inflammation through CD4^+^ lymphocyte recruitment [23, 33, 34]. However, perhaps more important than IL-17 production is their synthesis of GM-CSF. This cytokine supports attraction of monocytes and dendritic cells to the CNS and their activation, the mechanisms by which they contribute to neuroinflammation. GM-CSF furthermore acts as a positive feedback loop whereby it enhances the synthesis of IL-23 from antigen-presenting cells and so further sustains the activation and maturation of T_H_17 cells [35].

MS has been viewed historically as a CD4^+^ T cell-mediated autoimmune disease. However, the frequency of CD8^+^ T cells is greater than that of T helper cells in inflamed plaques, and CD8^+^ T cells show oligoclonal expansion in plaques, CSF, and blood which suggests they have a pathogenic role in MS too. Cytotoxic T cells destroy myelin by their perforin-granzyme mechanisms, resulting in the release of other autoantigens and circulus vitiosus continues [23, 36].

**Figure 2:**
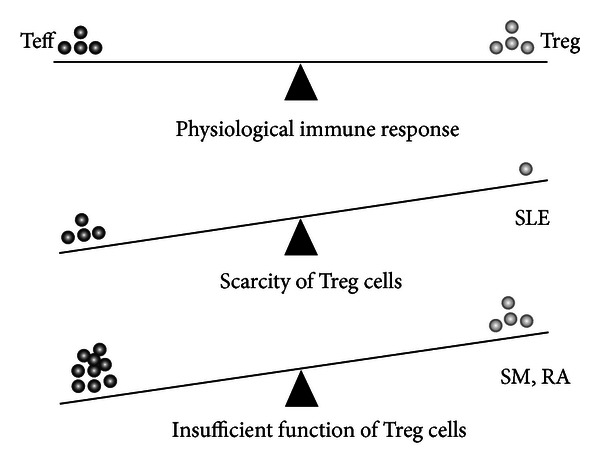
Causes of impaired Treg cells function in autoimmunity development. Failures of regulatory T (Treg) cell-mediated regulation can include: inadequate numbers of Treg cells owing to their inadequate development in the thymus, for example, due to a shortage of principal cytokines (IL-2, TGF-*β*) or costimulatory signals (CD28), and so forth. Further, the number of Treg cells can be in a physiological range; however, there are some defects in Treg-cell function that are intrinsic to Treg cells, for example, they do not synthesise sufficient quantity of immunosuppressive cytokines (IL-10, IL-35, and TGF-*β*), or there is a breakdown of their interaction with effector T cells. Ultimately, pathogenic effector T cells (Teff) are resistant to suppression by Treg cells owing to factors that are intrinsic to the effector cells or factors that are present in the inflammatory milieu that supports effector T cells resistance.

Regulatory T cells play a vital role in the regulation of immune processes. Based on the induction of autoimmune processes caused by the FOXP3 gene mutation, it was supposed that defective Treg cells might also contribute to the development of immunopathological processes in “more common” autoimmune disorders. This supposition has been confirmed. nTreg cells can contribute to the induction of autoimmunity by their decreased numbers, by the breakdown of their function, or simply by the reality that overactivated autoreactive T cells resist their immunosuppressive activities (Figure 2). The role of Treg cells in MS is rather controversial. While there have been reports on reduced frequency of nTreg cells in MS patients [37, 38], the majority of studies have found a similar frequency to the one observed in healthy individuals [39, 40]. However, several functional studies using *in vitro* suppression assays have documented impairments in Treg cells from MS patients [40–43]. What may be the cause for insufficient activities of nTreg cells in MS patients? It is probably a complex defect, such as reduced expression of coinhibitory molecules (CLTA-4, TIM-3, TIGIT) in their membranes, and insufficient synthesis of immunosuppressive cytokines [42, 44]. In this context, an interesting finding was reported by Schneider-Hohendorf et al. They disclosed an impaired migratory activity of Treg cells into the CNS in patients with relapsing-remitting MS (RR-MS) [45].

Adoptive transfer and depletion experiments in mice have also provided evidence that Treg can control the development and severity of EAE. For instance, in MOG-induced EAE, the transfer of Treg cells reduced disease severity and they were also able to suppress MOG-specific T cell responses *in vitro *[46]. In another study, in the PLP-induced model, the susceptibility of different mouse strains to EAE correlates inversely with the frequency of PLP-specific Treg cells [47]. These studies and others suggest that Treg cells influence the susceptibility to development of disease in the EAE models.

B cells do not cross the intact blood-brain barrier. However, once inflammation has started, they can enter the CNS. B cells, plasma cells, and myelin-specific antibodies are detected in MS plaques and in areas of active demyelination in MS patients [48, 49]. Recent studies have identified ectopic lymphoid follicles resembling germinal centres in the meninges [50–52]. It is possible that clonally expanded B cells, which originated in the meninges, may migrate to the parenchyma and participate in CNS damage. However, others did not confirm the findings [53], so the role of follicles remains controversial for the moment.

The intracerebral synthesis of IgG is typically oligoclonal; exact target antigens of these antibodies are, however, still elusive [23, 54, 55]. Antibodies can cause demyelination by opsonisation of myelin for phagocytosis or via complement activation. Besides the antibodies production, B cells have several antibody-independent functions. They include antigen presentation, T cell activation, and production of effector cytokines as reflected by introduction of anti-CD20 monoclonal antibodies to the treatment of MS patients (see later).

Ultimately, over previous decenniums, several authors have found NK cell defects in MS. It is, however, not yet known whether they are responsible for the development of the disease or only secondarily reflect the ongoing immunopathological process [35].

Until recently, neuromyelitis optica (NMO), also known as Devic's disease or Devic's syndrome, was considered a variation of multiple sclerosis. Now, it represents an independent disease, in which a person's own immune system attacks the optic nerves and spinal cord [32]. Although inflammation may also affect the brain, the lesions are different from those observed in MS. Unlike standard MS, the attacks are not mediated by the immune system's T cells, but rather by antibodies directed against aquaporin 4 (AQP4), a protein in the cell membranes of astrocytes. However, as antibodies belong to IgG1 class, their production requires T cells [56, 57]. Moreover, Varrin-Doyer et al. have brought evidence that T cells from NMO patients proliferated to intact AQP4 or AQP4 peptides [56]. 

Aquaporin 4 acts as a channel for transporting water across the cell membrane [58, 59]. In the processes of astrocytes that surround the BBB, a system responsible for preventing substances in the blood from crossing into the brain is found. It is currently unknown how the antibodies lead to demyelination. However, the induction of NMO seems to be resolved. Recently, some papers were published indicating the existence of structural homology and cross-reactivity between water channel proteins of *Helicobacter pylori* [60], *E. coli* aquaporin Z [61], and *Clostridium perfringens* adenosine triphosphate-binding cassette (ABC) transporter permease [56] and aquaporin 4, respectively.

Dominant cells that infiltrate the NMO lesions are neutrophils, the cells practically absent from lesions in MS. Their recruitment and activation can be mediated by IL-6, IL-8, and G-CSF. Levels of these cytokines were elevated in the cerebrospinal fluid (CSF) [62] as well as those of IL-17 [63]. 

Interferon beta (IFN-*β*), which has been used in the treatment of MS, must not be prescribed for the NMO treatment. Not only do patients exist who do not respond to the treatment, but moreover, IFN-*β* induces severe relapses and exacerbations of the disease in some of them [64, 65]. There is no cure for NMO. Currently azathioprine, prednisone, rituximab, cyclophosphamide, methotrexate, mitoxantrone, mycophenolate mofetil, intravenous immunoglobulins, or exchange plasmapheresis have been used for the treatment. However, recently it was shown that biological agents might be of some benefit in ameliorating a clinical status of the patients. New monoclonal antibodies, aquapuromab, were developed which also bind to AQP4, however to different epitopes as autoantibodies. Their attachment to AQP4 prevents pathogenic autoantibodies to bind to their targets because of steric hindrance and so to prevent their pathogenic activities. Aquaporumab activates neither the complement system, nor killer (K) cells, which prevents potential damage of target cells they bind with [66].

## 4. Biological Therapy of *Multiple Sclerosis*

A better understanding of the underlying mechanisms of MS found its reflection in the development of various immunotherapeutic agents. The first biologic agent used in the treatment of MS was IFN-*β* (1993), followed by glatiramer-acetate, monoclonal antibodies, FTY-720, and others. Each of them influences the ongoing immunopathogenic processes differently, trying to reestablish a previous physiological state (Table 1). However, it must be stressed that none of them has achieved its goal; all ameliorated the clinical state of treated patients only; no one was cured.

### 4.1. The First Line Agents for the Treatment of MS

Interferon beta is a cytokine with more immunomodulatory properties. It downregulates the expression of HLA class II molecules in antigen-presenting cells (APCs), which results in decreasing peptide presentation to T cells. On the contrary, it upregulates the expression of PDL-2 inhibitory molecules, which when interact with PD1 receptors in membranes of T cells, induce their apoptosis. IFN-*β* also inhibits proliferation of macrophages, resulting in reduction of their numbers and so activation of autoreactive T cells. Furthermore, IFN-*β* decreases also the transmigration of activated T cells into the CNS by the downregulation of their VLA-4 adhesive molecules, which are vital for binding to their VCAM-1 partners in membranes of endothelial cells [67].

IFN-*β* influences also activities of Treg cells. It upregulates the number of ligands for GITR receptors in membranes of dendritic cells. The interaction between GITR in Treg-cells and GITR-ligands in dendritic cells induces the proliferation of Treg lymphocytes, followed by an increase of their numbers and more active suppressive activities. The proliferation of Treg cells is further supported by other effect of IFN-*β*; it also downregulates the number of CTLA-4 molecules, which inhibit activities of Treg cells. This way, they become more susceptible for stimulatory cytokines, especially of IL-2, which is their basic homeostatic cytokine [68]. These experimental findings are corroborated by the results of the IFN-*β* treatment of MS patients with impaired nTreg function, which was shown to be reversed [41, 69].

Glatiramer acetate (GA) belongs to the first lineage of drugs used to treat MS. It is a random polymer of four amino acids found in myelin basic protein, namely, L-glutamic acid, L-lysine, L-alanine, and L-tyrosine. The mechanism of GA activity might be based on a blockade of grooves of HLA molecules. However, it seems that GA is endowed by immunomodulatory properties too. It was proved to induce, like IFN-*β*, the production of IL-1Ra, a natural inhibitor of IL-1, which results in inhibition of its proinflammatory activities. Furthermore, monocytes/macrophages under the GA activities produce less IL-1 and TNF, that is, the most potent proinflammatory cytokines and IL-12, the cytokine supporting polarisation of naïve T cells into the T_H_1 subset (Figure 1). On the contrary, it increases the synthesis of immunosuppressive IL-10 [70]. One supposes that GA-activated T cells enter the CNS and develop their anti-inflammatory and neuroprotective activities [71]. GA supports also suppressive activities of Treg cells by the upregulation of their coinhibitory molecules TIGIT and TIM-3 [42, 44, 72]. Like the treatment with IFN-*β*, that with GA resulted in reversal of impaired nTreg cells function [73].

### 4.2. Second-Line Agents for MS Treatment

Patients who are suboptimal responders to the standard immunomodulatory therapies are considered for treatment with second-line therapy represented by natalizumab and FTY720.

Natalizumab is a humanised monoclonal antibody against the cell adhesion VLA-4 molecule, its alpha 4 chain (VLA-4 belongs to beta-1 integrins: *α*4/*β*1). VLA-4 is located in membranes of T cells and its partner molecule is VCAM-1 in membranes of cerebral endothelial cells. This way, natalizumab prevents a transmigration of activated T cells into the CNS because they do not succeed in adhering to endothelial cells; macroscopically, this effect of natalizumab is perceived as lymphocytosis. Regulatory T cells are not affected by natalizumab; it influences neither their number nor function. Natalizumab reduces also the number of dendritic cells in the perivascular environment of the brain, indicating that its activity does not restrict itself to T cells only [74].

For some patients, discontinuation of the natalizumab treatment results in disease reactivation. Subjects who relapsed or had magnetic resonance imaging (MRI) worsening after treatment cessation had milder peripheral lymphocyte increases during the treatment. Furthermore, patients carrying a variant of the gene coding for Akt associated with reduced antiapoptotic efficiency (rs2498804T) had lower lymphocytosis and higher risk of disease reactivation [75, 76].

Natalizumab therapy may be associated with progressive multifocal leukoencephalopathy (PML), a potential life-threatening complication. PML is thought to be caused by reactivation of John Cunningham virus (JCV), primarily in the setting of immunosuppression [77]. Its pathological activity results in oligodendrocyte destruction [78]. An other complication of the natalizumab treatment is the induction of IRIS (immune reconstitution inflammatory syndrome), also known as the “immune recovery syndrome.” It is observed in some patients recovering from immunosuppression in whom the immune system begins to revive but then responds to a previously acquired opportunistic infections with an overwhelming inflammatory response that paradoxically makes the symptoms of the infection worse [78, 79]. Treg cells, rather induced than natural, probably take part in IRIS induction too because of inappropriate conditions for their induction. Nevertheless, natalizumab holds its position in the MS treatment when a physician considers the risk of the patient to develop PML or IRIS, and when his/her previous immunosuppressive treatment and a positivity of anti-JVC antibodies are taken into account.

*FTY720* (fingolimod), a derivative of myriocin, a fungal metabolite of the Chinese herb *Iscaria sinclarii*, is an other second-line immunomodulating drug approved for treating MS. It is a structural analogue of intracellular sphingosine that is phosphorylated by sphingosine kinase 2 *in vivo*. Fingolimod exerts its effect by mimicking sphingosine 1-phosphate (S1P) and the binding to four of five S1P receptors on lymphocytes results in their internalisation and prolonged downregulation. Without signals from S1P receptors, CD4^+^ and CD8^+^ T cells and B cells are unable to egress from secondary lymphoid tissues, resulting in a marked decrease of these cells in the periphery and their reduced recruitment to sites of inflammation. Approximately 80% of lymphocytes undergoes this reversible sequestration 3–5 hours after fingolimod application [80, 81].

Data on fingolimod effect on regulatory T cells are contradictory. There are reports claiming that it supports their proliferation and immunosuppressive activities although the mechanisms by which it exerts these effects are not reported [80]. The positive influence of fingolimod on Treg cells seems to be supported by clinical experience as its discontinuation in the treatment can result in relapse and induction of symptoms resembling IRIS [82, 83]. It can indicate that cessation of fingolimod treatment resulted also in reduction of Treg cells immunosuppressive activities and subsequently in reactivation of effector T cells.

On the other site, there are also reports showing fingolimod decreases activities of Treg cells. The way how fingolimod downregulates a Treg immunosuppressive potential is based on blocking IL-2-induced expansion, which is indispensable for their *in vivo* immunosuppressive activity [8, 84]. However, clinical experience connected with the above-mentioned relapse of the disease after fingolimod discontinuation does not support these results, or the results obtained in preclinical experiments do not always need to correlate with those when drugs are used in the real treatment of patients. It reminds of the events from 2006 when the superagonistic monoclonal antibodies anti-CD28 were applied to 4 volunteers. The antibodies supported the expansion of Treg cells in preclinical testing with mice; however, with the volunteers, they induced a cytokine storm and severe clinical symptoms threatening their lives [85].

### 4.3. Emerging Biological Agents for MS Treatment

The last decennium has brought the development of new biological agents that can modulate the MS disease processes, and we are now witnesses of many trials to verify their modes of action, benefits, and adverse reactions. Among them are novel monoclonal antibodies (mAb), especially anti-CD20, anti-CD52, and anti-CD25.

Anti-CD20 monoclonal antibodies bind to B cells and by activation of the complement system or killer cells, they destroy them. The rationale of a decrease of B cells for MS (and other autoimmune disorders) treatment is based on their other functions, not only those connected with production of antibodies. B cells belong to antigen-presenting cells too. They express HLA class II molecules, and engulfed protein antigens, previously bound to their immunoglobulin receptors, are then subsequently processed and bound to their grooves. The presentation of the “HLA-molecule - peptide” complex to T cells follows and by receiving costimulatory signals, T cells are activated [86, 87]. By destruction of B lymphocytes, anti-CD20 mAb reduce their number and so downregulate their ability to interact with autoreactive T cells, which results in attenuation of autoimmune processes. Concurrently, a cytokine profile in the microenvironment is changing in support of the induction and expansion of Treg cells [20, 88, 89]. Why was the CD20 molecule selected? The answer is relatively easy: CD20 is expressed on B cell lineage from the pre-B cell stage to the memory B cell stage, but not on plasma cells [90].

There are three different types of anti-CD20 mAb: rituximab, ocrelizumab, and ofatimumab. Rituximab and ofatumumab destroy B lymphocytes by the complement system activation, whereas ocrelizumab by antibody-dependent cellular cytotoxicity (ADCC), which is more advantageous as no proinflammatory fragments result from the complement activation. Furthermore, created apoptotic bodies are immediately engulfed by macrophages, also without any signs of inflammation induction [91].

Other two monoclonal antibodies have entered clinical trials: alemtuzumab, and daclizumab. Alemtuzumab* (Campath-1H)* is mAb-recognising CD52, the molecule expressed on T and B lymphocytes, natural killer (NK) cells, dendritic cells, monocytes, granulocytes, however, not on haematopoietic precursors. The biological role of CD52 seems to be in a participation of cell activation, at least in T lymphocytes. It was shown that CD52 cross-linking triggered their activation by induction of similar intracellular tyrosine phosphorylation events as employed by T cell receptor-mediated signalling. Furthermore, CD52 can serve as a costimulatory molecule involved in the induction of Treg cells [92, 93]. 

Treatment with alemtuzumab produces a very rapid and almost complete depletion of CD52-bearing cells in the circulation, mediated by ADCC [94, 95]. After depletion, repopulation of immune cells takes place differently. Monocytes return to normal values within three months; B cell counts return to baseline numbers also by three months and are then even increased to about 124% of pretreatment levels [91]. Increase of B cell counts is followed by enrichment in regulatory T cells. T cell counts recover much slower, as the depletion of CD4^+^ cells lasts a median of 61 months and of CD8^+^ cells for 30 months, respectively. The swift rise of B cells counts may explain a tendency of the alemtuzumab-treated patients to develop some autoimmune disorders, out of which the Graves' disease and autoimmune thrombocytopenia belong to the most severe [96, 97]. 

Alemtuzumab treatment of MS patients with relapsing-remitting forms of the disease has significantly reduced the risk of relapse and accumulation of disability, which suggested that it not only reduces disease activity due to the immune cell-depleting effect, but could perform other positive effects as well. Really, it was proved that it induced the production of neurotrophic factors in autoreactive T cells providing the CNS a neuroprotective effect. The group of Coles et al. showed that lymphocytes derived from alemtuzumab-treated MS patients produced enhanced amounts of brain-derived neurotrophic factor (BDNF) and ciliary neurotrophic factor (CNTF) upon antigen-specific stimulation with myelin basic protein (MBP) [98, 99].

Daclizumab is a humanised monoclonal antibody that binds to the alpha-chain of IL-2 receptor (CD25), thus effectively blocking the formation of its high-affinity form. Because the high-affinity IL-2 receptor signalling promotes the expansion of activated T cells *in vitro*, daclizumab was designed as a therapy that selectively inhibits T-cell activation and received approval as an add-on therapy to a standard immunosuppressive regimen for the prevention of acute allograft rejection in renal transplantation. Based on its mechanism of action, daclizumab represented an ideal therapy for T-cell-mediated autoimmune diseases too and was subsequently tested in the treatment of inflammatory uveitis and MS. In both of them, it significantly inhibited target organ inflammation. Subsequent studies of mechanisms of its action in MS resulted rather in a surprise; instead of inhibition of T-cell proliferation and production of cytokines, it was shown it had expanded and activated immunoregulatory CD56^bright^ NK cells, which gained access to the brain parenchyma and killed autologous activated T cells [100, 101].

Ultimately, regarding the role of lymphocytes in the induction and therapy of MS, one should also mention a possibility to induce and expand patient's own Treg cells *in vitro *and subsequently reintroduce them to the patient [102]. Treg cells could block both the initiation of autoimmune responses and inhibit the function of established autoreactive effector cells. The system was successfully tested in the EAE model of MS. The studies have disclosed that transfer of MBP-reactive Treg cells prevented disease when given prior to immunisation and prevented relapses when administered after the onset of disease. The effect was seen only when relevant myelin antigen-specific Treg cells were transferred, but not with polyclonal Treg cells [46, 103]. This could represent a stumbling block to the possible use of Treg therapy in MS, where the relevant antigens are not well defined. 

## 5. Laboratory Immunology and Clinical Practice

In recent years, we are witnesses of a substantial increase of our knowledge on particular immunopathological processes in MS, which has reflected in a better and more effective therapy that we shortly outlined in the previous paragraphs. However, there is still a question, which laboratory indicators should be taken into considerations when physicians are thinking over what type of treatment would fit best to a particular MS patient. The followup of particular population of T cells and their subsets in the peripheral blood surely informs a physician about response of the immune system to the therapy. For instance, level of expansion of CD56^bright^ NK cells and the decrease in ratios of T cells (as target cells) to CD56^bright^ NK cells (as effector cells) could represent a useful biomarker indicative of therapeutic response to daclizumab. However, our current knowledge of the great plasticity of T helper cells subsets and their ability of redifferentiation from one subset to another (e.g., T_H_2 to T_H_1, etc.) [104] will make us pay more attention to cytokines. If a cytokine profile is more tilted to a proinflammatory on the expense of an anti-inflammatory, it will indicate that the pathological process is more intensive and our therapy is less efficient. For instance, the ratio between anti-inflammatory IL-10 and proinflammatory IL-12 correlates with the disease activity, for example, if patients respond to the IFN-*β* treatment, the IL-10 to IL-12 ratio increases [105].

The monitoring of levels of some adhesive and costimulatory molecules (VLA-4, LFA-1, VCAM-1, CTLA-4, and TIM-3) follows the same objective. For instance, if an MS patient responds to IFN-*β* treatment, the levels of his/her adhesive molecules in the peripheral blood are decreasing [105]. How to treat it, when to change the therapy, and whether a drug combination should be used still remain upon the physician's discretion. Furthermore, MS is not a uniform disease; on the contrary, it has its own subtle differences based on the predominance of one type of immunopathological process over the other, which prevails differently in every MS patient. Obviously, the more we understand the underlying mechanisms and their interconnections, the more basic research will help physicians in their decisions.

## Figures and Tables

**Table 1:** Mechanisms of action of contemporary and emerging MS therapies.

Drug	Mechanism of action
IFN-*β* and Glatiramer acetate	Inhibition of the induction and proliferation of autoreactive T cells
Fingolimod	Prevention of egress of CD4^+ ^& CD8^+^ T cells, and B cells from secondary lymphoid tissues
Natalizumab	Blockade of transmigration of autoreactive T cells into the CNS
Rituximab	Depletion of B cells and attenuation of antibody independent proinflammatory B cell functions
Alemtuzumab	Depletion of CD4^+^ & CD8^+^ T cells, B cells, NK cells, and monocytes
Daclizumab	Expansion in CD56^bright ^NK cells; inhibition of activated T-cell proliferation

## List of Abbreviations

ADCC: Antibody-dependent cell-mediated cytolysis
APC: Antigen-presenting cells
BBB: Blood-brain barrier
BLIMP-1: B-lymphocyte-induced maturation protein
CMV: Cytomegalovirus
CTLA: Cytotoxic T lymphocyte antigen
DC: Dendritic cell
EAE: Experimental autoimmune encephalomyelitis
EBV: Epstein-Barr virus
FOXO: Forkhead box O
FOXP3: Forkhead box P
GITR: Glucocorticoid-induced tumour necrosis factor receptor family-related gene
GM-CSF: Granulocyte-macrophage colony-stimulating factor
GvHD: Graft versus host reaction disease
HBV: Hepatitis B virus
HLA: The major histocompatibility complex in man
HSV: Herpes simplex virus
IFN-*β*/IFN-*γ*: Interferon beta/gamma
IL: Interleukin
IL-1Ra: Interleukin 1 receptor antagonist
IRF4: Interferon regulatory factor 4
mAb: Monoclonal antibodies
MBP: Myelin basic protein
MOG: Myelin oligodendrocyte antigen
NK: Natural killer cells
PD: Programmed death
PLP: Proteolipid protein
STAT5: Signal transducer and activator of transcription 5
TCR: T-cell receptor
TIGIT: T-cell Ig and ITIM domain
TIM: T-cell immunoglobulin mucin protein.

## References

[1] Liu YJ (2006). A unified theory of central tolerance in the thymus. *Trends in Immunology*.

[2] Lan RY, Ansari AA, Lian ZX, Gershwin ME (2005). Regulatory T cells: development, function and role in autoimmunity. *Autoimmunity Reviews*.

[3] Sakaguchi S, Miyara M, Costantino CM, Hafler DA (2010). FOXP3 + regulatory T cells in the human immune system. *Nature Reviews Immunology*.

[4] Josefowicz SZ, Lu LF, Rudensky AY (2012). Regulatory T cells: mechanisms of differentiation and function. *Annual Review of Immunology*.

[5] Park SG, Mathur R, Long M (2010). T Regulatory cells maintain intestinal homeostasis by suppressing *γδ* T cells. *Immunity*.

[6] Ohkura N, Sakaguchi S (2011). Maturation of effector regulatory T cells. *Nature Immunology*.

[7] Ouyang W, Li MO (2011). Foxo: in command of T lymphocyte homeostasis and tolerance. *Trends in Immunology*.

[8] D’Cruz LM, Klein L (2005). Development and function of agonist-induced CD25^+^Foxp3^+^ regulatory T cells in the absence of interleukin 2 signaling. *Nature Immunology*.

[9] Bayer AL, Yu A, Adeegbe D, Malek TR (2005). Essential role for interleukin-2 for CD4^+^CD25^+^ T regulatory cell development during the neonatal period. *Journal of Experimental Medicine*.

[10] Setoguchi R, Hori S, Takahashi T, Sakaguchi S (2005). Homeostatic maintenance of natural Foxp3^+^ CD25^+^ CD4^+^ regulatory T cells by interleukin (IL)-2 and induction of autoimmune disease by IL-2 neutralization. *Journal of Experimental Medicine*.

[11] Langier S, Sade K, Kivity S (2010). Regulatory T cells: the suppressor arm of the immune system. *Autoimmunity Reviews*.

[12] Shevach EM (2009). Mechanisms of Foxp3^+^ T regulatory cell-mediated suppression. *Immunity*.

[13] Cvetanovich GL, Hafler DA (2010). Human regulatory T cells in autoimmune diseases. *Current Opinion in Immunology*.

[14] Tejera-Alhambra M, Alonso B, Teijeiro R, Ramos-Medina R, Aristimuno C, Valor L (2012). Perforin Expression by CD4^+^ Regulatory T Cells increases at multiple sclerosis relapse: sex differences. *International Journal of Molecular Sciences*.

[15] Hori S (2010). Developmental plasticity of Foxp3^+^ regulatory T cells. *Current Opinion in Immunology*.

[16] Josefowicz SZ, Niec RE, Kim HY, Treuting P, Chinen T, Zheng Y (2012). Extrathymically generated regulatory T cells control mucosal TH2 inflammation. *Nature*.

[17] Bilate AM, Lafaille JJ (2012). Induced CD4^+^Foxp3^+^ regulatory T cells in immune tolerance. *Annual Review of Immunology*.

[18] Knippenberg S, Peelen E, Smolders J, Thewissen M, Menheere P, Cohen Tervaert JW (2011). Reduction in IL-10 producing B cells (Breg) in multiple sclerosis is accompanied by a reduced naive/memory Breg ratio during a relapse but not in remission. *Journal of Neuroimmunology*.

[19] Weiss JM, Bilate AM, Gobert M, Ding Y, Curotto de Lafaille MA, Parkhurst CN (2012). Neuropilin 1 is expressed on thymus-derived natural regulatory T cells, but not mucosa-generated induced Foxp3^+^ T reg cells. *Journal of Experimental Medicine*.

[20] Lund FE, Randall TD (2010). Effector and regulatory B cells: modulators of CD4^+^ T cell immunity. *Nature Reviews Immunology*.

[21] Vitale G, Mion F, Pucillo C (2010). Regulatory B cells: evidence, developmental origin and population diversity. *Molecular Immunology*.

[22] Yoshizaki A, Miyagaki T, DiLillo DJ, Matsushita T, Horikawa M, Kountikov EI (2012). Regulatory B cells control T-cell autoimmunity through IL-21-dependent cognate interactions. *Nature*.

[23] Sospedra M, Martin R (2005). Immunology of multiple sclerosis. *Annual Review of Immunology*.

[24] Simon KC, Munger KL, Ascherio A (2012). Vitamin D and multiple sclerosis: epidemiology, immunology, and genetics. *Current Opinion in Neurology*.

[25] Baeke F, Takiishi T, Korf H, Gysemans C, Mathieu C (2010). Vitamin D: modulator of the immune system. *Current Opinion in Pharmacology*.

[26] Okada H, Khoury SJ (2012). Type17 T-cells in central nervous system autoimmunity and tumors. *Journal of Clinical Immunology*.

[27] Engelhardt B, Ransohoff RM (2012). Capture, crawl, cross: the T cell code to breach the blood-brain barriers. *Trends in Immunology*.

[28] Krumbholz M, Theil D, Steinmeyer F (2007). CCL19 is constitutively expressed in the CNS, up-regulated in neuroinflammation, active and also inactive multiple sclerosis lesions. *Journal of Neuroimmunology*.

[29] D'Agostino PM, Gottfried-Blackmore A, Anandasabapathy N, Bulloch K (2012). Brain dendritic cells: biology and pathology. *Acta Neuropathologica*.

[30] Becher B, Durell BG, Miga AV, Hickey WF, Noelle RJ (2001). The clinical course of experimental autoimmune encephalomyelitis and inflammation is controlled by the expression of CD40 within the central nervous system. *Journal of Experimental Medicine*.

[31] Vogel DY, Vereyken EJ, Glim JE, Heijnen PD, Moeton M, van der Valk P (2013). Macrophages in inflammatory multiple sclerosis lesions have an intermediate activation status. *Journal of Neuroinflammation*.

[32] O’Connor RA, Prendergast CT, Sabatos CA (2008). Cutting edge: Th1 cells facilitate the entry of Th17 cells to the central nervous system during experimental autoimmune encephalomyelitis. *Journal of Immunology*.

[33] Fletcher JM, Lalor SJ, Sweeney CM, Tubridy N, Mills KHG (2010). T cells in multiple sclerosis and experimental autoimmune encephalomyelitis. *Clinical and Experimental Immunology*.

[34] Kebir H, Kreymborg K, Ifergan I (2007). Human TH17 lymphocytes promote blood-brain barrier disruption and central nervous system inflammation. *Nature Medicine*.

[35] Buzzard KA, Broadley SA, Butzkueven H (2012). What do effective treatments for multiple sclerosis tell us about the molecular mechanisms involved in pathogenesis?. *International Journal of Molecular Sciences*.

[36] Friese MA, Fugger L (2009). Pathogenic CD8^+^ T cells in multiple sclerosis. *Annals of Neurology*.

[37] Huan J, Culbertson N, Spencer L (2005). Decreased FOXP3 levels in multiple sclerosis patients. *Journal of Neuroscience Research*.

[38] Praksova P, Stourac P, Bednarik J, Vlckova E, Mikulkova Z, Michalek J (2012). Immunoregulatory T cells in multiple sclerosis and the effect of interferon beta and glatiramer acetate treatment on T cell subpopulations. *Journal of the Neurological Sciences*.

[39] Feger U, Luther C, Poeschel S, Melms A, Tolosa E, Wiendl H (2007). Increased frequency of CD4^+^ CD25^+^ regulatory T cells in the cerebrospinal fluid but not in the blood of multiple sclerosis patients. *Clinical and Experimental Immunology*.

[40] Haas J, Hug A, Viehöver A (2005). Reduced suppressive effect of CD4^+^CD25high regulatory T cells on the T cell immune response against myelin oligodendrocyte glycoprotein in patients with multiple sclerosis. *European Journal of Immunology*.

[41] Kumar M, Putzki N, Limmroth V (2006). CD4^+^CD25^+^Foxp3^+^ T lymphocytes fail to suppress myelin basic protein-induced proliferation in patients with multiple sclerosis. *Journal of Neuroimmunology*.

[42] Lowther DE, Hafler DA (2012). Regulatory T cells in the central nervous system. *Immunological Reviews*.

[43] Buckner JH (2010). Mechanisms of impaired regulation by CD4^+^ CD25^+^ Foxp3^+^ regulatory T cells in human autoimmune diseases. *Nature Reviews Immunology*.

[44] Joller N, Peters A, Anderson AC, Kuchroo VK (2012). Immune checkpoints in central nervous system autoimmunity. *Immunological Reviews*.

[45] Schneider-Hohendorf T, Stenner MP, Weidenfeller C (2010). Regulatory T cells exhibit enhanced migratory characteristics, a feature impaired in patients with multiple sclerosis. *European Journal of Immunology*.

[46] Kohm AP, Carpentier PA, Anger HA, Miller SD (2002). Cutting edge: CD4^+^CD25^+^ regulatory T cells suppress antigen-specific autoreactive immune responses and central nervous system inflammation during active experimental autoimmune encephalomyelitis. *Journal of Immunology*.

[47] Reddy J, Illes Z, Zhang X (2004). Myelin proteolipid protein-specific CD4^+^CD25^+^ regulatory cells mediate genetic resistance to experimental autoimmune encephalomyelitis. *Proceedings of the National Academy of Sciences of the United States of America*.

[48] Esiri MM (1977). Immunoglobulin containing cells in multiple sclerosis plaques. *Lancet*.

[49] Genain CP, Cannella B, Hauser SL, Raine CS (1999). Identification of autoantibodies associated with myelin damage in multiple sclerosis. *Nature Medicine*.

[50] Lovato L, Willis SN, Rodig SJ (2011). Related B cell clones populate the meninges and parenchyma of patients with multiple sclerosis. *Brain*.

[51] Magliozzi R, Howell O, Vora A (2007). Meningeal B-cell follicles in secondary progressive multiple sclerosis associate with early onset of disease and severe cortical pathology. *Brain*.

[52] Serafini B, Rosicarelli B, Magliozzi R, Stigliano E, Aloisi F (2004). Detection of ectopic B-cell follicles with germinal centers in the meninges of patients with secondary progressive multiple sclerosis. *Brain Pathology*.

[53] Kooi EJ, Geurts JJ, van Horssen J, Bø L, van der Valk P (2009). Meningeal inflammation is not associated with cortical demyelination in chronic multiple sclerosis. *Journal of Neuropathology and Experimental Neurology*.

[54] Nylander A, Hafler DA (2012). Multiple sclerosis. *Journal of Clinical Investigation*.

[55] Peelen E, Damoiseaux J, Smolders J, Knippenberg S, Menheere P, Tervaert JW (2011). Th17 expansion in MS patients is counterbalanced by an expanded CD39+ regulatory T cell population during remission but not during relapse. *Journal of Neuroimmunology*.

[56] Varrin-Doyer M, Spencer CM, Schulze-Topphoff U, Nelson PA, Stroud RM, Cree BA (2012). Aquaporin 4-specific T cells in neuromyelitis optica exhibit a Th17 bias and recognize Clostridium ABC transporter. *Annals of Neurology*.

[57] Nurieva RI, Chung Y (2010). Understanding the development and function of T follicular helper cells. *Cellular and Molecular Immunology*.

[58] Lennon PVA, Wingerchuk DM, Kryzer TJ (2004). A serum autoantibody marker of neuromyelitis optica: distinction from multiple sclerosis. *Lancet*.

[59] Jarius S, Paul F, Franciotta D (2008). Mechanisms of disease: aquaporin-4 antibodies in neuromyelitis optica. *Nature Clinical Practice Neurology*.

[60] Li W, Minohara M, Piao H (2009). Association of anti-Helicobacter pylori neutrophil-activating protein antibody response with anti-aquaporin-4 autoimmunity in Japanese patients with multiple sclerosis and neuromyelitis optica. *Multiple Sclerosis*.

[61] Ren Z, Wang Y, Duan T, Patel J, Liggett T, Loda E (2012). Cross-immunoreactivity between bacterial aquaporin-Z and human aquaporin-4: potential relevance to neuromyelitis optica. *Journal of Immunology*.

[62] Uzawa A, Mori M, Arai K (2010). Cytokine and chemokine profiles in neuromyelitis optica: significance of interleukin-6. *Multiple Sclerosis*.

[63] Wang HH, Dai YQ, Qiu W, Lu ZQ, Peng FH, Wang YG (2011). Interleukin-17-secreting T cells in neuromyelitis optica and multiple sclerosis during relapse. *Journal of Clinical Neuroscience*.

[64] Papadopoulos MC, Verkman AS (2012). Aquaporin 4 and neuromyelitis optica. *Lancet Neurology*.

[65] Palace J, Leite MI, Nairne A, Vincent A (2010). Interferon beta treatment in neuromyelitis optica: increase in relapses and aquaporin 4 antibody titers. *Archives of Neurology*.

[66] Tradtrantip L, Zhang H, Saadoun S, Phuan PW, Lam C, Papadopoulos MC (2012). Anti-aquaporin-4 monoclonal antibody blocker therapy for neuromyelitis optica. *Annals of Neurology*.

[67] Graber JJ, McGraw CA, Kimbrough D, Dhib-Jalbut S (2010). Overlapping and distinct mechanisms of action of multiple sclerosis therapies. *Clinical Neurology and Neurosurgery*.

[68] Chen M, Chen G, Deng S, Liu X, Hutton GJ (2012). IFN-beta induces the proliferation of CD4^+^CD25^+^Foxp3^+^ regulatory T cells through upregulation of GITRL on dendritic cells in the treatment of multiple sclerosis. *Journal of Neuroimmunology*.

[69] de Andrés C, Aristimuño C, de las Heras V (2007). Interferon beta-1a therapy enhances CD4^+^ regulatory T-cell function: an ex vivo and in vitro longitudinal study in relapsing-remitting multiple sclerosis. *Journal of Neuroimmunology*.

[70] Burger D, Molnarfi N, Weber MS (2009). Glatiramer acetate increases IL-1 receptor antagonist but decreases T cell-induced IL-1*β* in human monocytes and multiple sclerosis. *Proceedings of the National Academy of Sciences of the United States of America*.

[71] Lalive PH, Neuhaus O, Benkhoucha M (2011). Glatiramer acetate in the treatment of multiple sclerosis: emerging concepts regarding its mechanism of action. *CNS Drugs*.

[72] Haas J, Korporal M, Balint B, Fritzsching B, Schwarz A, Wildemann B (2009). Glatiramer acetate improves regulatory T-cell function by expansion of naive CD4^+^CD25^+^Foxp3^+^CD31+ T-cells in patients with multiple sclerosis. *Journal of Neuroimmunology*.

[73] Hong J, Li N, Zhang X, Zheng B, Zhang JZ (2005). Induction of CD4^+^CD25^+^ regulatory T cells by copolymer-I through activation of transcription factor Foxp3. *Proceedings of the National Academy of Sciences of the United States of America*.

[74] Skarica M, Eckstein C, Whartenby KA, Calabresi PA (2011). Novel mechanisms of immune modulation of natalizumab in multiple sclerosis patients. *Journal of Neuroimmunology*.

[75] Martelli AM, Tabellini G, Bressanin D, Ognibene A, Goto K, Cocco L (2012). The emerging multiple roles of nuclear Akt. *Biochimica et Biophysica Acta*.

[76] Rossi S, Motta C, Studer V, Monteleone F, De Chiara V, Buttari F (2013). A genetic variant of the anti-apoptotic protein Akt predicts natalizumab-induced lymphocytosis and post-natalizumab multiple sclerosis reactivation. *Multiple Sclerosis*.

[77] Mancini N, Clementi M, Burioni R (2012). Natalizumab-associated progressive multifocal leukoencephalopathy. *New England Journal of Medicine*.

[78] Chataway J, Miller DH (2013). Natalizumab therapy for multiple sclerosis. *Neurotherapeutics*.

[79] Shelburne SA, Visnegarwala F, Darcourt J (2005). Incidence and risk factors for immune reconstitution inflammatory syndrome during highly active antiretroviral therapy. *AIDS*.

[80] Sawicka E, Dubois G, Jarai G (2005). The sphingosine 1-phosphate receptor agonist FTY720 differentially affects the sequestration of CD4^+^/CD25^+^ T-regulatory cells and enhances their functional activity. *Journal of Immunology*.

[81] Lee CW, Choi JW, Chun J (2010). Neurologyogical S1P signaling as an emerging mechanism of action of oral FTY720 (Fingolimod) in multiple sclerosis. *Archives of Pharmacal Research*.

[82] Rigau V, Mania A, Befort P, Carlander B, Jonquet O, Lassmann H (2012). Lethal multiple sclerosis relapse after natalizumab withdrawal. *Neurology*.

[83] Marousi S, Travasarou M, Karageorgiou CE, Gheuens S, Koralnik IJ (2012). Simultaneous Pml-Iris after discontinuation of natalizumab in a patient with MS. *Neurology*.

[84] Wolf AM, Eller K, Zeiser R (2009). The sphingosine 1-phosphate receptor agonist FTY720 potently inhibits regulatory T cell proliferation in vitro and in vivo. *Journal of Immunology*.

[85] Suntharalingam G, Perry MR, Ward S (2006). Cytokine storm in a phase 1 trial of the anti-CD28 monoclonal antibody TGN1412. *New England Journal of Medicine*.

[86] Buc M (1995). The major histocompatibility complex in man. *Folia Biologica*.

[87] Benesova Y, Vasku A, Stourac P, Hladikova M, Fiala A, Bednarik J (2013). Association of HLA-DRB1*1501 tagging rs3135388 gene polymorphism with multiple sclerosis. *Journal of Neuroimmunology*.

[88] Barun B, Bar-Or A (2012). Treatment of multiple sclerosis with anti-CD20 antibodies. *Clinical Immunology*.

[89] Ray A, Basu S, Williams CB, Salzman NH, Dittel BN (2012). A novel IL-10-independent regulatory role for B cells in suppressing autoimmunity by maintenance of regulatory T cells via GITR ligand. *Journal of Immunology*.

[90] Stashenko P, Nadler LM, Hardy R, Schlossman SF (1980). Characterization of a human B lymphocyte-specific antigen. *Journal of Immunology*.

[91] Saidha S, Eckstein C, Calabresi PA (2012). New and emerging disease modifying therapies for multiple sclerosis. *Annals of the New York Academy of Sciences*.

[92] Rowan WC, Hale G, Tite JP, Brett SJ (1995). Cross-linking of the CAMPATH-1 antigen (CD52) triggers activation of normal human T lymphocytes. *International Immunology*.

[93] Hederer RA, Guntermann C, Miller N (2000). The CD45 tyrosine phosphatase regulates Campath-1H (CD52)-induced TCR-dependent signal transduction in human T cells. *International Immunology*.

[94] Coles AJ, Cox A, Le Page E (2006). The window of therapeutic opportunity in multiple sclerosis: evidence from monoclonal antibody therapy. *Journal of Neurology*.

[95] Coles AJ, Compston DAS, Selmaj KW (2008). Alemtuzumab versus interferon beta-1a in early multiple sclerosis. *New England Journal of Medicine*.

[96] Thompson SAJ, Jones JL, Cox AL, Compston DAS, Coles AJ (2010). B-Cell reconstitution and BAFF after alemtuzumab (Campath-1H) treatment of multiple sclerosis. *Journal of Clinical Immunology*.

[97] Coles AJ, Wing M, Smith S (1999). Pulsed monoclonal antibody treatment and autoimmune thyroid disease in multiple sclerosis. *Lancet*.

[98] Jones JL, Anderson JM, Phuah CL (2010). Improvement in disability after alemtuzumab treatment of multiple sclerosis is associated with neuroprotective autoimmunity. *Brain*.

[99] Klotz L, Meuth SG, Wiendl H (2012). Immune mechanisms of new therapeutic strategies in multiple sclerosis-A focus on alemtuzumab. *Clinical Immunology*.

[100] Bielekova B (2013). Daclizumab therapy for multiple sclerosis. *Neurotherapeutics*.

[101] Martin R (2012). Anti-CD25 (daclizumab) monoclonal antibody therapy in relapsing-remitting multiple sclerosis. *Clinical Immunology*.

[102] Brusko T, Bluestone J (2008). Clinical application of regulatory T cells for treatment of type 1 diabetes and transplantation. *European Journal of Immunology*.

[103] Stephens LA, Malpass KH, Anderton SM (2009). Curing CNS autoimmune disease with myelin-reactive Foxp3^+^ Treg. *European Journal of Immunology*.

[104] Nakayamada S, Takahashi H, Kanno Y, O'Shea JJ (2012). Helper T cell diversity and plasticity. *Current Opinion in Immunology*.

[105] Graber JJ, Dhib-Jalbut S (2011). Biomarkers of disease activity in multiple sclerosis. *Journal of the Neurological Sciences*.

